# Intestinal Colonization With *Bifidobacterium longum* Subspecies Is Associated With Length at Birth, Exclusive Breastfeeding, and Decreased Risk of Enteric Virus Infections, but Not With Histo-Blood Group Antigens, Oral Vaccine Response or Later Growth in Three Birth Cohorts

**DOI:** 10.3389/fped.2022.804798

**Published:** 2022-02-16

**Authors:** Josh M. Colston, Mami Taniuchi, Tahmina Ahmed, Tania Ferdousi, Furqan Kabir, Estomih Mduma, Rosemary Nshama, Najeeha Talat Iqbal, Rashidul Haque, Tahmeed Ahmed, Zulfiqar Ali Bhutta, Margaret N. Kosek, James A. Platts-Mills

**Affiliations:** ^1^Division of Infectious Diseases and International Health, University of Virginia, Charlottesville, VA, United States; ^2^International Centre for Diarrhoeal Disease Research, Bangladesh, Dhaka, Bangladesh; ^3^Department of Pediatrics and Child Health, Aga Khan University, Karachi, Pakistan; ^4^Haydom Global Health Research Centre, Haydom, Tanzania; ^5^Department of Public Health Sciences, University of Virginia School of Medicine, Charlottesville, VA, United States

**Keywords:** *Bifidobacteria*, infant nutrition, microbiome, cohort study, global health

## Abstract

*Bifidobacterium longum* subspecies detected in infant stool have been associated with numerous subsequent health outcomes and are potential early markers of deviation from healthy developmental trajectories. This analysis derived indicators of carriage and early colonization with *B. infantis* and *B. longum* and quantified their associations with a panel of early-life exposures and outcomes. In a sub-study nested within a multi-site birth cohort, extant stool samples from infants in Bangladesh, Pakistan and Tanzania were tested for presence and quantity of two *Bifidobacterium longum* subspecies. The results were matched to indicators of nutritional status, enteropathogen infection, histo-blood group antigens, vaccine response and feeding status and regression models were fitted to test for associations while adjusting for covariates. *B. infantis* was associated with lower quantity of and decreased odds of colonization with *B. longum*, and vice versa. Length at birth was associated with a 0.36 increase in log_10_ *B. infantis* and a 0.28 decrease in *B. longum* quantity at 1 month of age. *B. infantis* colonization was associated with fewer viral infections and small reductions in the risk of rotavirus and sapovirus infections, but not reduced overall diarrheal disease risk. No associations with vaccine responses, HBGAs or later nutritional status were identified. Suboptimal intrauterine growth and a shorter duration of exclusive breastfeeding may predispose infants to early intestinal colonization with the *B. longum* subspecies at the expense of *B. infantis*, thus denying them potential benefits of reduced enteric virus episodes.

## Introduction

The first 2 years of life are a critical period for metabolic and immunological development, during which birth mode, feeding practices, dietary intake, antibiotic use, histo-blood group antigen (HBGA) genes, and enteropathogen exposure all interact to have lasting impacts on an infant's health and nutritional prospects (1–3). Recently, links have been identified between gut microbiota maturation and undernutrition, enteropathogen infection, diarrheal disease and oral vaccine response (1, 2, 4). In particular, certain species and subspecies of the *Bifidobacterium* genus of bacteria, gram-positive anaerobes that proliferate in the mammalian gastrointestinal tract, are thought to be particularly beneficial for gut homeostasis, immunomodulation, nutritional status, and protection against diarrhea and pathogens (5–8). *Bifidobacterium* species colonize the human gut very rapidly following birth, becoming the predominant intestinal microbiota by early infancy and remaining an abundant presence in the microbiome into adulthood (6, 9). The *infantis* subspecies [initially thought to be its own species, but now classified as a subspecies of *Bifidobacterium longum* (10)] is a particularly successful colonizer in many low income settings, tending to thrive in the microbiome of breastfed infants, due to being well-adapted to harvest carbon from human milk oligosaccharides (HMOs) (9, 11, 12). The quantity of *B. longum* subsp. *infantis* (*B. infantis*) detected in infant fecal samples has been positively associated with improved weight gain, thymic development, gut health and response to oral vaccines (4, 11, 13, 14), however colonization of the gut by this bacteria subspecies can be delayed or disrupted in infants who are pre-term, not optimally breastfed, whose mothers are non-secretors (fucosyltransferase2-negative), or for whom HMO intake is otherwise constrained (11, 15–18). *B. infantis* and other *B. longum* subspecies have therefore come under consideration as potential early markers of deviation from healthy developmental trajectories, and as targets for interventions promoting early colonization, such as probiotics and breastfeeding promotion, particularly in low resource settings where oral vaccine efficacy is low and rates of undernutrition highest (4, 11, 13, 19, 20). However, to date, baseline rates of *Bifidobacterium* carriage have not been well-described in such populations and there is a need for research linking colonization with dietary, morbidity and environmental data longitudinally (17).

In this sub-study, nested within a multi-site birth cohort, stool samples from infants in three low-resource communities were tested for *B. infantis* and *B. longum* subsp. *longum* (*B. longum*). Indicators of carriage and early colonization were derived, described, and included in regression models to quantify their associations with a panel of early-life exposures and outcomes. The a-priori hypothesis to be tested was that subjects that were already colonized with *B. infantis* and had higher levels of carriage at 1 month of age would have improved nutritional, vaccine response and enteropathogen risk profiles in ways that would not be true of *B. longum*.

## Materials and Methods

### Study Population

This sub-study was nested within the Etiology Risk Factors and Interactions of Enteric Infections and Malnutrition and the Consequences for Child Health and Development (MAL-ED) project at three of that parent study's sites in Bangladesh, Pakistan and Tanzania, which have been described previously (21–23). For the parent study, subjects were recruited into cohorts at birth according to inclusion criteria described previously (21) and monitored continuously over their first 2 years from 2009 to 2014. The original MAL-ED cohorts included data from 265 subjects in Bangladesh, 277 in Pakistan and 262 in Tanzania, a total of 804 individuals across the three sites (24). Subjects were included in this sub-study if they met both of the following criteria: (1) Remained in the original MAL-ED cohort for the full 24 months of follow-up; (2) Contributed stool samples at all three-monthly assessments from 1 to 3 months of age inclusive. The Johns Hopkins Institutional Review Board gave ethical approval for MAL-ED as did partner institutions at each site. Written informed consent was obtained from the caregivers of all participating children.

### *Bifidobacteria* Colonization

DNA was extracted from extant stool samples collected from MAL-ED subjects at 1, 2, and 3 months of age using the QIAamp DNA Stool Mini Kit (Qiagen, Gaithersburg, MD) with a modified manufacturer's protocol described previously (25). DNA specimens were stored at −80°C before being analyzed for the presence and quantity of the two subspecies using quantitative polymerase chain reaction (qPCR) previously described (20, 26). All detections with a cycle threshold ≥40 were considered negative.

### Other Variables

The following variables were matched to the *Bifidobacterium* results by MAL-ED subject and included in the analysis based on documented or hypothesized potential associations with *Bifidobacterium* colonization:

#### Nutritional Status

Anthropometric data from the parent MAL-ED study was compiled giving the subjects' length in centimeters and weight in kilograms at birth and measured at monthly assessments as described elsewhere (27). Length- and weight-for-age *Z*-scores (LAZ, WAZ) were calculated for each subject at each available anthropometric assessment based on their length/height, weight and age using the WHO Child Growth Standards STATA igrowup package, with implausible values recoded as missing (28). LAZ values were not available from the Pakistan site.

#### Enteropathogen Infection

Stool samples collected at monthly intervals and during caregiver-reported diarrheal episodes had previously been tested for the presence of numerous enteropathogen species using qPCR, enzyme-linked immunosorbent assay (ELISA), and microscopy diagnostics as part of the parent MAL-ED study, the methods and findings of which have been described extensively elsewhere (29–31). Results from qPCR were preferentially used where available; otherwise, results from other methods were substituted. Infection status for each of 13 highly prevalent or endemic enteric pathogen species or pathotypes were treated as binary outcome variables as was infection status for any of the three pathogen taxa - viruses, bacteria, and protozoa. The pathogens included were adenovirus, astrovirus, norovirus genogroup GII, rotavirus, sapovirus, *Campylobacter jejuni* or *coli*, enteroaggregative *Escherichia coli* (*E. coli*) (EAEC), typical enteropathogenic *E. coli* (EPEC), heat-labile enterotoxigenic *E. coli* (LT-ETEC), and heat-stable ETEC (ST-ETEC), *Shigella*/enteroinvasive *E. coli* (EIEC) (qPCR uses the same gene target for these two closely related pathogens), *Cryptosporidium*, and *Giardia*. Samples from the same subject that were positive for the same pathogen were considered discrete infection episodes if separated either by an intermediate negative sample or a period of 14 days, with the exception of *Campylobacter* spp. and norovirus, for which a period of 30 days was used, and the two protozoa for which three intermediate negative samples were required [criteria previously documented by Colston et al. (3)]. Diarrhea episodes were also included.

#### HBGA Status

Secretor (FUT2) status and Lewis (FUT3) type were ascertained for study subjects and their mothers from saliva samples using a phenotyping assay in Bangladesh and sequencing of the FUT2 and FUT3 genes in Tanzania according to methods documented previously (3). These variables were not ascertained at the Pakistan site.

#### Vaccine Response

Serum neutralizing antibody log_2_ titers for poliovirus, tetanus and pertussis were quantified by IgG ELISA on blood samples collected at a target age of 15 months (32).

#### Feeding Status

Daily information on exclusive breastfeeding was ascertained by caregiver report during weekly home visits.

#### Socio-Economic Status

The WAMI-index, a composite indicator of socio-economic status that is valid for comparisons in multi-country studies (33), was calculated for all MAL-ED subjects based on a baseline questionnaire.

### Statistical Methods

Variability in *Bifidobacterium* colonization among the subjects was visualized by plotting the density of samples by cycle threshold (Ct) value by subspecies, site, and month of age. Then two indicators of *B. longum* subspecies colonization were constructed. As a continuous indicator of *Bifidobacterium* carriage, the relative quantity (40-Ct) in the 1-month samples was log-transformed with base 10, a method previously used to analyze pathogen quantity in stool samples (31). As a binary indicator of early colonization, the Ct values at 1 month of age were dichotomized at a value of 30 (determined based on visualization of the Ct distributions) to divide the samples into those with low (Ct ≥ 30) compared to high (Ct <30) carriage. For comparison, all analyses were performed using both colonization measures for both subspecies.

Initially, the colonization indicators were treated as time-fixed outcomes in regression models (linear for quantity, logistic for early colonization) fitted to each of a series of exposures manifest in early infancy, namely LAZ and WAZ-scores at enrollment (shortly after birth), the proportion of days in the first month of life on which the subject was exclusively breastfed and FUT2 and FUT3 status of both the children and their mothers. In a second stage, the colonization indicators were treated as time-fixed exposures and fitted in linear regression models to outcomes ascertained in later infancy, namely LAZ and WAZ-score at 24 months of age, cumulative number of viral, bacterial, and protozoal infections from 1 to 24 months of age, and log_2_ tetanus, pertussis, and polio 1 titers at 15 months of age. Finally, the colonization indicators were fitted in longitudinal models to time-varying outcomes ascertained at multiple time points from 1 to 24 months of age, namely LAZ and WAZ-scores, diarrheal episodes, and infection episodes for each enteropathogen. Generalized linear models (GLMs) with cluster-robust variance estimation were used with Gaussian family specified for the two, continuous anthropometric outcomes, and with Poisson family for the binary, infection status outcome [a modified Poisson regression approach, with which coefficient estimates can be interpreted as the log of risk ratio (RRs) estimates (34, 35)]. For the diarrheal episodes outcome, Cox proportional hazards models were fitted treating the subjects' age as survival time, reporting of a diarrheal episode as failure events and allowing for multiple failures per subject (3). All models were adjusted for site, sex, and WAMI-index, the longitudinal models were also adjusted for WAZ-score at enrollment and breastfeeding status (exclusive vs. non-exclusive by day of follow-up), and the GLMs included linear, cubic, and quadratic terms for the subjects' age in continuous months. Analyses were carried out using Stata 16 (36) and R 3.6.2 (37).

## Results

**Figure 1:**
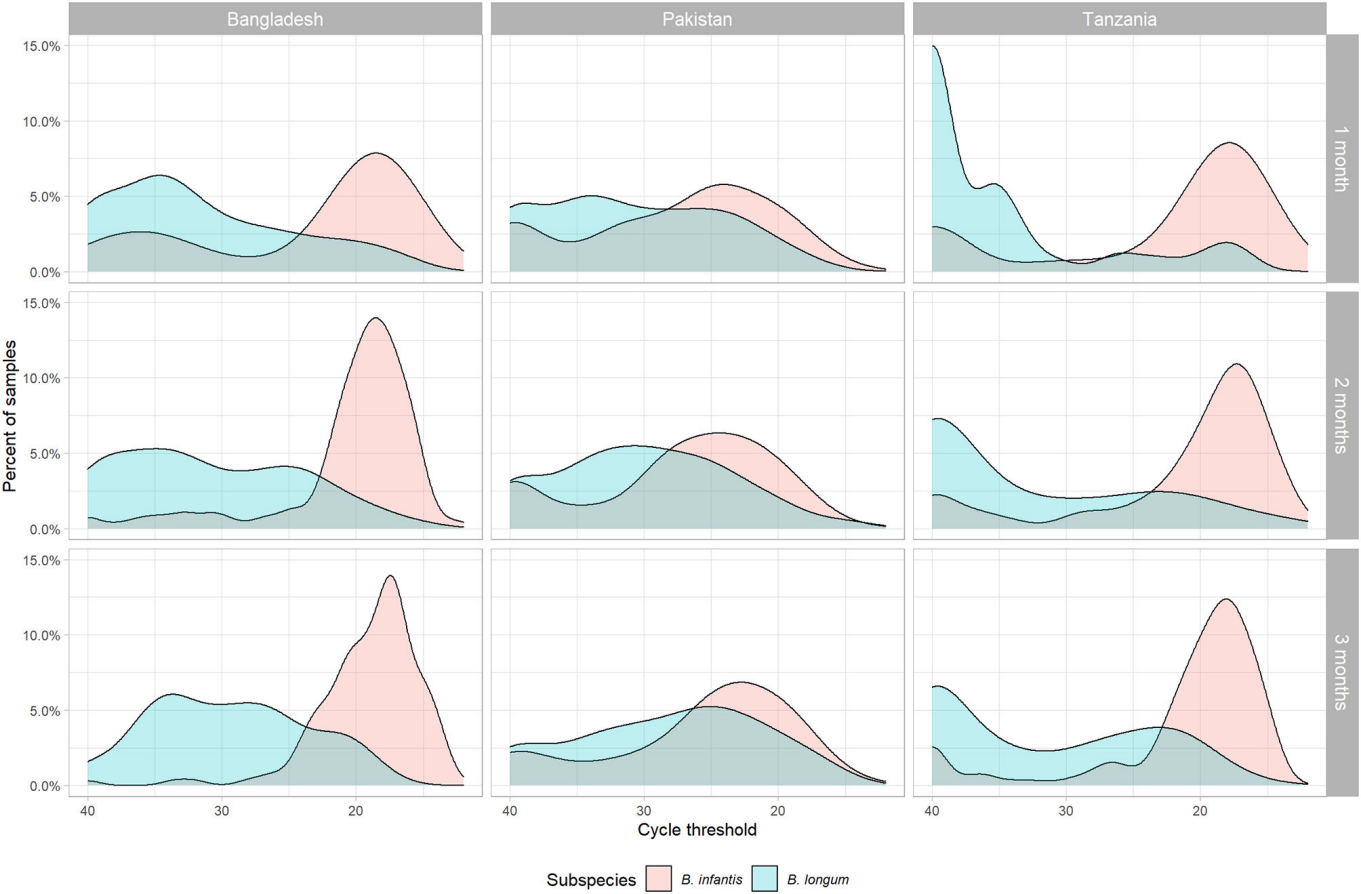
Density distribution of cycle threshold values for two subspecies of *Bifidobacterium longum* (*infantis* and *longum*) measured by PCR in stool samples by subspecies, site, and month of age.

Four hundred and seventy-two of the original 804 subjects met the criteria for inclusion in this sub-study (Table 1), including 189 subjects in Bangladesh (71.3% of the original cohort), 180 in Pakistan (65.0%), and 103 in Tanzania (39.3%). The relatively low rate of inclusion in Tanzania was due to there being fewer available 1-month samples at that site. The distribution of B. infantis quantity followed a bimodal distribution at 1, 2, and 3 months of age, with the majority of children at all sites having high Ct values (Figure 1) and a subset having a low or undetectable quantity. The modal B. infantis Ct value was fairly constant within sites across the age points, while between-site differences were also small. B. longum tended to have a wider distribution of Ct values than B. infantis with a higher modal value which, in Pakistan and, most notably, Tanzania showed a tendency to decrease with increasing age.

**Table 1:** Summary statistics for variables included in the analysis by study site and overall.

	**Bangladesh**	**Pakistan**	**Tanzania**	**Total**
**Total subjects**	**189**	**180**	**103**	**472**
***B. infantis***				
Early colonization^a^	137 (72.5)	116 (64.4)	79 (76.7)	332 (70.3)
log_10_ quantity	4.9 (2.5)	3.6 (2.2)	5.1 (2.7)	4.5 (2.5)
***B. longum***				
Early colonization^a^	65 (34.4)	77 (42.8)	17 (16.5)	159 (33.7)
log_10_ quantity	2.5 (2.0)	2.7 (2.0)	1.4 (2.1)	2.4 (2.1)
Enrollment LAZ	−1.0 (1.1)	–	−0.9 (1.1)	−1.0 (1.1)
Enrollment WAZ	−1.2 (0.9)	−1.4 (1.0)	−0.1 (1.0)	−1.0 (1.1)
Proportion of days exclusively breastfed	0.9 (0.2)	0.2 (0.2)	0.6 (0.3)	0.6 (0.4)
WAMI	0.5 (0.1)	0.5 (0.2)	0.2 (0.1)	0.4 (0.2)
Child's secretor status^a^	134 (70.9)	–	59 (57.3)	193 (40.9)
Child's Lewis type^a^	144 (76.2)	–	74 (71.8)	218 (46.2)
Mother's secretor status^a^	128 (67.7)	–	62 (60.2)	190 (40.3)
Mother's Lewis type^a^	142 (75.1)	–	61 (59.2)	203 (43.0)
LAZ at 24 months	−2.1 (0.9)	–	−2.6 (1.0)	−2.2 (1.0)
WAZ at 24 months	−1.6 (1.0)	−1.6 (1.0)	−1.2 (1.0)	−1.6 (1.0)
log2 tetanus titer - 15 months	2.4 (2.3)	2.4 (2.2)	3.5 (2.0)	2.6 (2.2)
log2 pertussis titer - 15 months	9.3 (1.8)	9.5 (1.7)	9.1 (2.5)	9.4 (1.9)
log2 polio 1 titer - 15 months	8.5 (2.7)	6.2 (4.1)	8.6 (2.1)	7.5 (3.5)
Diarrhea episodes^b^	7.2 (5.3)	8.6 (6.8)	0.6 (0.9)	6.3 (6.2)
**Enteropathogen infection episodes^b^**				
Adenovirus 40/41	5.9 (2.5)	3.1 (2.0)	1.7 (1.3)	3.9 (2.7)
Astrovirus	5.1 (2.0)	3.4 (1.7)	1.3 (1.3)	3.6 (2.3)
Norovirus GII	5.4 (2.3)	5.6 (2.4)	3.3 (1.5)	5.0 (2.4)
Rotavirus	2.5 (1.8)	1.1 (1.0)	1.1 (1.2)	1.7 (1.6)
Sapovirus	4.9 (2.1)	4.2 (1.9)	2.2 (1.2)	4.0 (2.1)
*C. jejuni/coli*	9.0 (3.4)	7.9 (4.0)	8.8 (2.7)	8.5 (3.5)
EAEC	10.3 (2.7)	8.7 (3.1)	13.0 (2.9)	10.3 (3.3)
Typical EPEC	5.1 (2.2)	2.6 (1.6)	4.0 (2.1)	3.9 (2.2)
LT-ETEC	3.9 (1.9)	2.3 (1.6)	5.4 (2.4)	3.6 (2.2)
ST-ETEC	8.3 (3.0)	2.4 (1.5)	5.9 (2.6)	5.5 (3.6)
*Shigella* spp./EIEC	4.3 (2.4)	1.9 (1.5)	3.9 (2.1)	3.3 (2.3)
*Cryptosporidium* spp.	1.7 (1.3)	1.6 (1.2)	2.1 (1.1)	1.7 (1.2)
*Giardia* spp.	3.1 (2.2)	6.6 (2.3)	4.5 (2.2)	4.8 (2.7)

*Unless otherwise indicated, values are mean (and standard deviation).*

a *Summary statistics for binary variables are number (%).*

b *Summary statistics for diarrhea and infection episodes are mean (and standard deviation) of within-subject total discrete episodes from 0 to 2 years*.

Prevalence of early *B. infantis* colonization ranged from just under two thirds of subjects having attained high (Ct <30) carriage by the first month of life in Pakistan, to over three quarters in Tanzania (Table 1). The mean log_10_ relative quantities followed a similar pattern across the sites. Prevalence of *B. longum* colonization at 1-month was much lower overall and the ranking of sites was reversed compared with *B. infantis*, only surpassing 40% in the Pakistan site and with fewer than one in six 1-month-olds colonized in Tanzania. Similarly, relative *B. longum* quantity was lower and the ranking of sites by mean quantity reversed compared with *B. infantis*. The other variables have been described elsewhere, but notably the proportion of days exclusively breastfed varied considerably from just 0.2 in Pakistan to 0.9 in Bangladesh (3, 24, 33, 38–40).

Coefficient estimates from regression models of the associations between the time-fixed, early-life exposures and the four indicators of *Bifidobacterium* colonization (Table 2) indicate that a one unit increase in enrollment LAZ-score was associated with a 0.36 increase in log_10_ *B. infantis* and a 0.28 decrease in *B. longum* quantity at 1 month of age. Correspondingly, the odds of early *B. infantis* colonization increased by 41%, while the odds of early *B. longum* decreased by 26% for each one-unit LAZ-score increase. Equivalent effect estimates for WAZ-score were all in the same direction but non-significant. Subjects who were exclusively breastfed for the first 3 months of life had an estimated 1.21 reduction in *B. longum* quantity and an 81% decreased odds of early *B. longum* colonization compared to those who had never been exclusively breastfed, but equivalent effect estimates for *B. infantis* were not statistically significant. No significant effect estimates were observed between any of the four maternal or child HBGA status variables and colonization with either subspecies. Early *B. infantis* colonization was statistically significantly associated with a 46% decreased odds of early *B. longum* colonization (and vice versa), and with a 0.69 decrease in *B. longum* quantity.

**Table 2:** Coefficient estimates (with 95% confidence intervals) from regression models of the associations between seven time-fixed, early-life exposures and indicators of quantity (linear regression) and early colonization (logistic regression) for two *Bifidobacterium longum* subspecies, adjusted for site, socio-economic status (WAMI score) and sex.

**Exposure**	***B. infantis***		***B. longum***	
	**log_**10**_ quantity**	**Early colonization**	**log_**10**_ quantity**	**Early colonization**
Enrollment LAZ-score	0.36^*^ (0.09, 0.64)	1.41^**^ (1.10, 1.81)	−0.28^*^ (−0.50, −0.06)	0.74^*^ (0.58, 0.95)
Enrollment WAZ-score	0.15 (−0.08, 0.37)	1.14 (0.93, 1.39)	−0.12 (−0.31, 0.06)	0.88 (0.72, 1.07)
Proportion of days exclusively breastfed	−0.09 (−1.10, 0.92)	0.89 (0.35, 2.25)	−1.21^**^ (−2.05, −0.37)	0.19^**^ (0.07, 0.51)
Child secretor positive	0.16 (−0.64, 0.96)	1.21 (0.61, 2.39)	−0.43 (−1.07, 0.21)	0.52 (0.27, 1.00)
Child Lewis positive	−0.24 (−1.23, 0.75)	0.99 (0.43, 2.28)	−0.10 (−0.90, 0.70)	1.14 (0.49, 2.66)
Mother secretor positive	−0.76 (−1.64, 0.12)	0.49 (0.20, 1.18)	−0.13 (−0.84, 0.58)	0.77 (0.36, 1.66)
Mother Lewis positive	0.61 (−0.67, 1.90)	1.65 (0.60, 4.53)	0.47 (−0.56, 1.50)	1.71 (0.52, 5.56)
***B. infantis***				
log10 quantity	–	–	−0.11^*^ (−0.22, −0.00)	0.94 (0.87, 1.02)
Early colonization	–	–	−0.69^***^ (−1.10, −0.29)	0.54^**^ (0.36, 0.83)
***B. longum***				
log10 quantity	−0.11^*^ (−0.21, −0.00)	0.85^***^ (0.77, 0.93)	–	–
Early colonization	−0.35 (−0.83, 0.12)	0.54^**^ (0.36, 0.83)	–	–

*** *p < 0.001,*

** *p = 0.001–0.01,*

* *p = 0.01–0.05*.

Coefficient estimates from linear regression models that treated each of the *Bifidobacterium* colonization variables in turn as time-fixed exposures and variables ascertained in later childhood as outcomes are shown in Table 3. No statistically significant effects of colonization at 1 month were observed on anthropometry at 24 months, cumulative bacterial and protozoal infections from 1 to 24 months, or vaccine response at 15 months with the exception of a slight reduction in log_2_ polio 1 titer with increases in *B. infantis* quantity. A 1 log_10_ increase in *B. infantis* quantity measured at 1 month was also slightly statistically significantly associated with a 0.26 decrease, and early *B. infantis* colonization with a 1.24 decrease, in the cumulative number of enteric viral infections experienced from 1 to 24 months, but no equivalent effects on this outcome were observed for *B. longum*.

**Table 3:** Coefficient estimates (with 95% confidence intervals) from time-fixed linear regression models of the associations between indicators of quantity and early colonization for two *Bifidobacterium longum* subspecies and eight outcomes in later childhood adjusted for site, WAZ-score at enrollment, socio-economic status (WAMI score) and sex.

**Outcome**	***B. infantis***		***B. longum***	
	**log_**10**_ quantity**	**Early colonization**	**log_**10**_ quantity**	**Early colonization**
LAZ-score at 24 months	0.03 (−0.00, 0.07)	0.17 (−0.07, 0.40)	−0.00 (−0.05, 0.05)	−0.07 (−0.30, 0.16)
WAZ-score at 24 months	−0.01 (−0.04, 0.03)	−0.06 (−0.24, 0.13)	−0.01 (−0.05, 0.04)	−0.00 (−0.19, 0.18)
Number of viral infections	−0.26^*^ (−0.47, −0.04)	−1.24^*^ (−2.39, −0.09)	0.02 (−0.24, 0.27)	0.05 (−1.09, 1.17)
Number of bacterial infections	0.14 (−0.21, 0.50)	0.35 (−1.53, 2.24)	−0.15 (−0.58, 0.27)	−0.23 (−2.07, 1.61)
Number of protozoan infections	0.02 (−0.08, 0.12)	−0.02 (−0.56, 0.51)	−0.02 (−0.14, 0.10)	0.04 (−0.48, 0.57)
log_2_ tetanus titer - 15 months	0.07 (−0.07, 0.20)	0.66 (−0.05, 1.37)	0.09 (−0.07, 0.26)	0.06 (−0.63, 0.75)
log_2_ pertussis titer - 15 months	−0.02 (−0.11, 0.07)	0.15 (−0.32, 0.62)	0.10 (−0.01, 0.21)	0.26 (−0.19, 0.72)
log_2_ polio 1 titer - 15 months	−0.08^*^ (−0.16, −0.00)	−0.21 (−0.62, 0.19)	−0.02 (−0.11, 0.07)	−0.25 (−0.64, 0.14)

^***^p < 0.001, **p = 0.001–0.01,

* *p = 0.01–0.05*.

Equivalent results from longitudinal models of the associations between the *Bifidobacterium* colonization indicators and time-varying anthropometric and enteric infection outcomes from ages 1–24 months are shown in Table 4. A 1 log_10_ increase in *B. infantis* quantity at 1 month of age was associated with a 4% decrease in the relative risk of rotavirus and a 3% decrease in sapovirus risk between 1 and 24 months of age. The equivalent effects of early *B. infantis* colonization were, respectively, a 19% and a 16% decrease in relative risk of infection. *B. infantis* quantity was also statistically significantly associated with a 2% increase in the risk of a diarrheal episode and a 3% increase in *Campylobacter jejuni*/*coli* infection, but the equivalent estimates for early *B. infantis* colonization were not statistically significant. No statistically significant effects were observed of *B. longum* colonization on any of the time-varying outcomes—with the exception of a 4% decrease in *Cryptosporidium* infection—or of *B. infantis* on the anthropometric outcomes or protozoal infections.

**Table 4:** Coefficient estimates and risk ratios (with 95% confidence intervals) from longitudinal models of the associations between indicators of quantity and early colonization for two *Bifidobacterium longum* subspecies and time-varying anthropometric and enteric infection outcomes from ages 1–24 months adjusted for site, age, feeding status, WAZ-score at enrollment, socio-economic status (WAMI score) and sex.

**Outcome**	***B. infantis***		***B. longum***	
	**log_**10**_ quantity**	**Early colonization**	**log_**10**_ quantity**	**Early colonization**
LAZ-score	0.02 (−0.02, 0.06)	0.12 (−0.10, 0.35)	−0.02 (−0.07, 0.02)	−0.12 (−0.32, 0.08)
WAZ-score	0.02 (−0.02, 0.06)	0.07 (−0.16, 0.30)	−0.01 (−0.06, 0.04)	−0.07 (−0.29, 0.16)
Diarrhea episodes	1.02^*^ (1.00, 1.04)	1.05 (0.96, 1.14)	1.00 (0.98, 1.02)	0.95 (0.88, 1.03)
**Enteropathogen infection episodes**				
Adenovirus 40/41	0.99 (0.97, 1.02)	0.99 (0.87, 1.12)	0.98 (0.96, 1.01)	0.96 (0.85, 1.08)
Astrovirus	0.99 (0.97, 1.01)	0.97 (0.87, 1.09)	1.00 (0.97, 1.02)	0.99 (0.89, 1.09)
Norovirus GII	0.99 (0.97, 1.02)	1.00 (0.89, 1.13)	1.00 (0.97, 1.02)	0.96 (0.85, 1.08)
Rotavirus	0.96^**^ (0.93, 0.99)	0.81^*^ (0.69, 0.96)	1.00 (0.97, 1.04)	0.99 (0.84, 1.15)
Sapovirus	0.97^**^ (0.95, 0.99)	0.84^**^ (0.76, 0.93)	1.02 (1.00, 1.04)	1.09 (0.98, 1.21)
*C. jejuni/coli*	1.03^*^ (1.00, 1.06)	1.16 (0.99, 1.36)	1.00 (0.96, 1.03)	0.99 (0.84, 1.16)
EAEC	0.99 (0.97, 1.01)	0.96 (0.86, 1.08)	0.99 (0.97, 1.02)	0.99 (0.89, 1.10)
Typical EPEC	0.99 (0.97, 1.02)	0.99 (0.88, 1.11)	0.99 (0.97, 1.02)	1.01 (0.91, 1.13)
LT-ETEC	0.99 (0.97, 1.02)	0.98 (0.87, 1.11)	1.02 (0.99, 1.05)	1.11 (0.97, 1.26)
ST-ETEC	0.99 (0.97, 1.01)	0.97 (0.86, 1.09)	0.99 (0.96, 1.01)	0.98 (0.87, 1.10)
*Shigella*/EIEC	1.01 (0.98, 1.04)	1.02 (0.87, 1.19)	0.99 (0.96, 1.03)	0.98 (0.85, 1.14)
*Cryptosporidium*	1.01 (0.98, 1.04)	1.00 (0.86, 1.17)	0.96^*^ (0.93, 0.99)	0.89 (0.76, 1.04)
*Giardia*	1.00 (0.96, 1.04)	1.00 (0.82, 1.22)	1.02 (0.98, 1.06)	1.12 (0.92, 1.35)

^***^p < 0.001,

** *p = 0.001–0.01,*

* *p = 0.01–0.05*.

## Discussion

*Bifidobacterium longum* subspecies are a ubiquitous presence in the gut microbiota of human infants, transmitted vertically from the mother at birth via vaginal delivery or shortly after in the first breastmilk meals and thereupon proliferating by metabolizing the HMOs consumed through subsequent breastfeeding (41, 42). Metabolites produced by this fermentation process, such as acetate, formate and lactate, promote epithelial barrier function (43) and create an acidic environment hostile to bacterial pathogens (44), while the adhesion of enterocytes to bifidobacterial interacting with pilis and other surface-associated cellular structures trigger some of the first proinflammatory responses, priming the immune system for early development (8, 41, 45). These processes are believed to underly the well-documented associations of intestinal bifidobacterial colonization with numerous health benefits later in infancy and beyond (41). Dietary probiotics are increasingly being introduced to correct gut microbiota dysbiosis and treat or prevent diarrhea in infancy and its sequalae such as growth faltering (46). *B. infantis* is increasingly a candidate for probiotic intervention with commercially available products such as EVC001 (20), since early colonization with the subspecies is thought to lead to improved growth and vaccine response, fewer enteric infections, and protection against later diarrheal disease. However, most published information from low-resource settings is from Bangladesh, and little is known about normal carriage rates of the bacterium in other such contexts. Since infant microbiota composition varies from place to place, there is a need for longitudinal data from diverse settings that includes detailed dietary intake and other factors (17). With this study, we contribute evidence from two other such locations—Tanzania and Pakistan—in addition to Bangladesh, quantifying colonization rates and associations between two *Bifidobacterium* subspecies and both early-life exposures and infection, growth, and vaccine outcomes in later childhood.

We confirm that early *B. infantis* colonization is common but not universal across these three diverse settings, occurring in between two thirds and three quarters of infants by 1 month of age. Early colonization with *B. longum* is less common, with only around a third of 1 month-old infants overall having high carriage. Among the strongest associations identified by this analysis were the negative relationships between colonization with the two subspecies, observed across both the quantity and early colonization variables and at both site- and subject-level. That the detection of *B. infantis* was associated with lower quantity of and decreased odds of colonization with *B. longum*, and vice versa suggests competition between the two subspecies, perhaps for HMO harvesting.

The main enteropathogen-specific effect observed was a reduction in the number of viral infections conferred by *B. infantis* colonization, and specifically, a decrease in the risk of rotavirus and sapovirus infection comparable in magnitude to the previously documented protective effects of secretor status (3), improved sanitation (47) and hydrometeorological factors (48). These findings are consistent with the hypothesis that the *infantis* subspecies plays a role in the priming of early cellular immunity (7), however, this did not translate into a decrease in overall risk of diarrheal disease—indeed, this risk was slightly increased in subjects with higher *B. infantis* quantity. No clear associations between *infantis* colonization and humoral vaccine responses were detected, in contrast with previously published findings from Bangladesh (9), but in line with those for rotavirus vaccine immunogenicity in Zimbabwe (49). Furthermore, a slight increase in risk of *Campylobacter jejuni*/*coli* was observed for each log_10_ increase in *B. infantis* quantity, in contrast to previous findings from a Peruvian cohort which found *Bifidobacterium* amplicon sequence variant abundance to be associated with slightly lower *Campylobacter* burden at 6 months of age (50). We also report for the first time a small protective effect of *B. longum* quantity on *Cryptosporidium* infection risk.

We also did not find evidence in support of the hypothesis that *B. infantis* colonization in early infancy promotes improvements in nutritional status later in childhood, however length at birth was associated with increased *B. infantis* and reduced *B. longum* colonization. Nor did these findings confirm links between maternal and child HBGA status and *Bifidobacterium* colonization at 1 month. It is also notable that we did not find an association of prolonged exclusive breastfeeding with increased *B. infantis* but we did with decreased quantity of and odds of early colonization with *B. longum*. While rates of exclusive breastfeeding varied widely between the three sites, results from site-specific analyses were consistent with this pooled result (apart from in Bangladesh, the one site with near universal exclusive breastfeeding).

This study was subject to several limitations, which should be considered when interpreting the findings. Firstly, using as an inclusion criteria for the sub-study that the subjects must have completed 24 months of follow-up with three available stool samples in the first 3 months of life may introduce selection bias by excluding subjects from socio-economically precarious households who may differ systematically with respect to the associations analyzed. Secondly, it has recently been reported that, even within the *B. infantis* subspecies, there is a considerable diversity of strains exhibiting heterogeneity in their repertoire of HMO utilization genes (12). It is possible that, since the broad subspecies assays used here were insufficiently sensitive to distinguish HMO-utilizing from non-utilizing strains within the *B. infantis* subspecies, the true effect of breastmilk specifically on those exhibiting the HMO utilization phenotype were masked and instead only indirectly evident in the suppression of *B. longum* colonization. Future research should aim to distinguish among *B. infantis* subspecies strains on the basis of genotypes associated with HMO utilization such as H5 positivity (12). Further research is needed to determine whether shaping the infant flora toward these subspecies through widespread administration of probiotics such as *B. infantis* EVC001 would be as beneficial a population health intervention as has been hypothesized (20).

In conclusion, taken as a whole these findings suggest that infants with suboptimal intrauterine growth, manifested as shorter length at birth, and a shorter duration of exclusive breastfeeding may be predisposed to early intestinal colonization with the *B. longum* subspecies at the expense of *B. infantis*. Such infants may thereby be denied the potential benefits of *B. infantis* colonization such as reduced enteric virus episodes. However, the effects of the identified associations were small in magnitude, and previously documented benefits of *Bifidobacterium* colonization on later nutritional status were not replicated in this sub-study.

## Data Availability Statement

The raw data supporting the conclusions of this article will be made available upon reasonable request to the corresponding author, without undue reservation.

## Ethics Statement

Ethical approval for the “MAL-ED” project was given by the Johns Hopkins Institutional Review Board as well as from the respective partner institutions for each site including: The Institutional Review Board for Health Science Research of the University of Virginia; The Ethical Review Committee of ICDDR, B; The Ethics Review Committee at the Aga Khan University; The Medical Research Coordinating Committee of the National Institute of Medical Research, Tanzania; The Ministry of Health and Social Welfare of Tanzania. Written informed consent to participate in this study wasprovided by the participants' legal guardian/next of kin.

## Author Contributions

JC carried out the data analysis, drafted the initial manuscript, reviewed, and revised the final manuscript. TahmeA, RH, ZB, and EM designed the data collection instruments, oversaw the fieldwork and data collection, reviewed, and revised the manuscript. MT oversaw and TF, FK, NI, RN, and TahmiA carried out the laboratory analysis. MK critically reviewed the manuscript for important intellectual content. JP-M conceptualized and designed the study, reviewed, and revised the manuscript. All authors approved the final manuscript as submitted and agreed to be accountable for all aspects of the work.

## Funding

The Etiology, Risk Factors, and Interactions of Enteric Infections and Malnutrition and the Consequences for Child Health and Development Project (MAL-ED) is carried out as a collaborative project supported by the Bill & Melinda Gates Foundation (BMFG 47075), the Foundation for the National Institutes of Health, and the National Institutes of Health, Fogarty International Center. Additional diagnostics for this sub-study was also supported by BMFG (INV-000372) to JP-M.

## Conflict of Interest

The authors declare that the research was conducted in the absence of any commercial or financial relationships that could be construed as a potential conflict of interest.

## Publisher's Note

All claims expressed in this article are solely those of the authors and do not necessarily represent those of their affiliated organizations, or those of the publisher, the editors and the reviewers. Any product that may be evaluated in this article, or claim that may be made by its manufacturer, is not guaranteed or endorsed by the publisher.
